# Substrate binding accelerates the conformational transitions and substrate dissociation in multidrug efflux transporter AcrB

**DOI:** 10.3389/fmicb.2015.00302

**Published:** 2015-04-13

**Authors:** Beibei Wang, Jingwei Weng, Wenning Wang

**Affiliations:** ^1^Shanghai Key Laboratory of Molecular Catalysis and Innovative Materials, Department of Chemistry, Fudan UniversityShanghai, China; ^2^Institutes of Biomedical Sciences, Fudan UniversityShanghai, China

**Keywords:** AcrB, MD simulation, substrate binding, allosteric effect, drug extrusion, positive cooperativity

## Abstract

The tripartite efflux pump assembly AcrAB-TolC is the major multidrug resistance transporter in *E. coli.* The inner membrane transporter AcrB is a homotrimer, energized by the proton movement down the transmembrane electrochemical gradient. The asymmetric crystal structures of AcrB with three monomers in distinct conformational states [access (A), binding (B) and extrusion (E)] support a functional rotating mechanism, in which each monomer of AcrB cycles among the three states in a concerted way. However, the relationship between the conformational changes during functional rotation and drug translocation has not been totally understood. Here, we explored the conformational changes of the AcrB homotrimer during the ABE to BEA transition in different substrate-binding states using targeted MD simulations. It was found that the dissociation of substrate from the distal binding pocket of B monomer is closely related to the concerted conformational changes in the translocation pathway, especially the side chain reorientation of Phe628 and Tyr327. A second substrate binding at the proximal binding pocket of A monomer evidently accelerates the conformational transitions as well as substrate dissociation in B monomer. The acceleration effect of the multi-substrate binding mode provides a molecular explanation for the positive cooperativity observed in the kinetic studies of substrate efflux and deepens our understanding of the functional rotating mechanism of AcrB.

## Introduction

In Gram-negative bacteria, resistance-nodulation-division (RND) superfamily proteins play a major role in the efflux of a wide range of antibiotics and toxic compounds out of cell. RND superfamily, together with other classes of multidrug efflux pumps, constitute one of the major mechanisms of multidrug resistance (MDR) in bacteria, which represents a serious impediment to improved healthcare (Higgins, 2007). RND transporter is embedded in the inner membrane and works as a drug-proton antiporter. Fueled by proton diffusion down the transmembrane (TM) electrochemical gradient, it collects substrates from the periplasm or the inner leaflet of inner membrane and extrudes them to the lumen of outer membrane component (Nikaido, 1996). The RND transporter AcrB in *Escherichia coli* has been extensively studied as a prototype of the family, which exports a number of dyes, detergents, chloramphenicol, tetracyclines, macrolides, β-lactams, fluoroquinolones, and organic solvents. It functions in the form of AcrAB-TolC tripartite complex (Zgurskaya and Nikaido, 1999b; Tikhonova and Zgurskaya, 2004; Collu et al., 2012; Du et al., 2014) by working collaboratively with the periplasmic adaptor protein AcrA (Zgurskaya and Nikaido, 1999a; Mikolosko et al., 2006) and the outer membrane protein TolC (Koronakis et al., 2000). As the core of the complex, AcrB is responsible for substrate recognition and energy supplement (Elkins and Nikaido, 2002).

**Figure 1:**
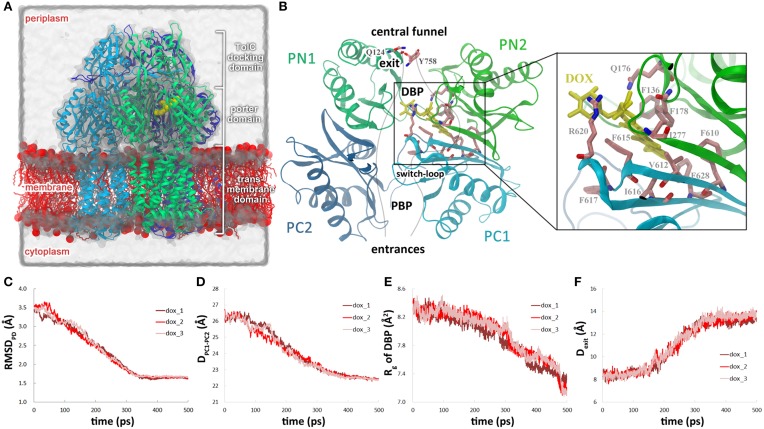
**Conformational changes of the translocation pathway in the dox system. (A)** Simulation system of AcrB trimer buried in POPE bilayer and solvated with water molecules. A, B, and E monomers of AcrB in cartoon representation are colored in cyan, green and blue, respectively. Doxorubicin bound in the DBP of B monomer is shown in the VDW representation (yellow). Lipid molecules are colored in red with the charged head groups shown as balls and the hydrophobic tails as sticks. Some lipid molecules are removed for clarity. **(B)** A schematic view of the substrate translocation pathway in B monomer (gray lines). Subdomains PC1, PC2, PN1, and PN2 are colored in blue, cyan, dark green, and green, respectively. The residues lining the DBP is represented by the licorice mode and colored by atom type (C: salmon, O: red, N: blue). Residues Qln124 and Tyr758 that form the exit gate are also indicated. **(C)** Variation of the C_α_ root-mean-square deviation (RMSD) of the porter domain in B monomer relative to the target structure. **(D)** Variations of the distance between the mass centers of PC1 and PC2 subdomains. **(E)** Variations of the radius of gyration of DBP in B monomer. **(F)** Variations of the Gln124-Tyr758 inter-residue distance at the exit gate. The coordinate of each residue is represented by its C_α_ atom. For simplicity, we use the targeted MD simulation time to describe the conformational transition events in the text, but it is worth noting that the simulation time only denotes the relative progress of the targeted MD trajectories and is not necessarily proportional to actual time.

Crystallographic studies have revealed the structure of AcrB as a homotrimer (Murakami et al., 2002, 2006; Seeger et al., 2006). Each monomer is composed of a TM domain, a porter domain and a TolC-docking domain (Figure 1A). The TM domain contains 12 TM helices and encompasses a putative proton relay pathway lined by Asp407 and Asp408 on TM4 helix, Lys940 on TM10 helix and Thr978 and Arg971 on TM11 helix, which harvests proton motive force from the transmembrane electrochemical gradient (Murakami et al., 2006; Seeger et al., 2006, 2009; Su et al., 2006; Takatsuka and Nikaido, 2006). The porter domain and the TolC docking domain are folded by two periplasmic loops of the TM domain, one between TM1 and TM2 helices and the other between TM7 and TM8 helices. The porter domain could be further divided into four subdomains PN1, PN2, PC1, and PC2 (Figure 1B), the inter-domain space between which forms the pathway for substrate translocation (Sennhauser et al., 2007; Husain and Nikaido, 2010; Nakashima et al., 2011; Yao et al., 2013). Despite of the diversity of entrances (Husain and Nikaido, 2010; Nakashima et al., 2011; Yao et al., 2013), substrates are found to constantly pass through the cleft between PC1 and PC2, the “switch-loop” (also called Phe-617 loop, G-loop), the cavity enclosed by PC1, PN1, and PN2, and the exit constricted by Gln124 (on PN1 subdomain) and Tyr758 (on the TolC-docking domain) (Figure 1B) during the translocation process, and finally enter the central funnel enclosed by the TolC-docking domains. Two binding pockets have been identified along the translocation pathway inside the porter domain. The pocket more distal from the entrances, i.e., the distal binding pocket (DBP), lies between PC1 and PN2 and is rich in aromatic residues including Phe610, Phe615, Phe617, and Phe628 on PC1 and Phe136 and Phe178 on PN2 (Figure 1B) (Murakami et al., 2006; Nakashima et al., 2011; Eicher et al., 2012). The proximal binding pocket (PBP, also called access pocket) between PC1 and PC2 is more hydrophilic (Figure 1B), capturing substrates through a combination of hydrogen bond, hydrophobic and π −π stacking interactions (Nakashima et al., 2011; Eicher et al., 2012).

AcrB is highly dynamic and its conformational changes are essential for its transport activity (Takatsuka and Nikaido, 2007, 2009; Seeger et al., 2008). Three major conformational states of monomers have been identified in the asymmetric crystal structures (Murakami et al., 2006; Seeger et al., 2006; Sennhauser et al., 2007; Nakashima et al., 2011; Eicher et al., 2012). The access (A, also called loose, L) and binding (B, also called tight, T) states both have an opened PC1/PC2 cleft and an occluded exit, but differs in the binding pockets. The DBP in B state is intact and capable of accommodating substrates, whereas in A state, the DBP partially collapses and the PBP is found capable of substrate binding instead (Nakashima et al., 2011; Eicher et al., 2012). The extrusion (E, also called open, O) state adopts a closed PC1/PC2 cleft and an opened exit for substrate extrusion. Edified by the asymmetric crystal structures, each monomer of AcrB trimer is believed to undergo a succession of transitions among the three states during transportation, cycling from A to B to E and back to A state, i.e., the functional rotating mechanism (Murakami et al., 2006; Seeger et al., 2006; Pos, 2009). The mechanism also predicts that the transition of each monomer is dependent on the conformations of its neighbors. When A monomer (originally staying in A state) evolves to B state, B and E monomers would respectively switch into E and A states simultaneously, accomplishing one ABE→BEA transition step.

The functional rotating mechanism implicates highly cooperative conformational changes among the three monomers. Subsequent kinetic studies indeed revealed positive cooperativity in substrate efflux (Nagano and Nikaido, 2009; Lim and Nikaido, 2010). However, the underlying detailed picture of the functional rotating and the cooperativity in substrate efflux remain elusive, especially the relationship between the conformational changes in AcrB and the kinetic behavior of substrate efflux. Molecular dynamics (MD) simulation is a powerful tool in providing high spatiotemporal resolution details for conformational changes of proteins. MD simulations have been used to reveal the dynamics of RND transporters (Fischer and Kandt, 2013; Yamane et al., 2013), the movement of substrate (Fischer and Kandt, 2011; Feng et al., 2012) and the interaction network between the transporter and substrate (Vargiu and Nikaido, 2012; Kinana et al., 2013). Due to the sampling efficiency problem of the conventional MD simulation, some enhanced sampling protocols, such as targeted MD (Ma and Karplus, 1997; Kong et al., 2002; van der Vaart et al., 2004; Compoint et al., 2005; Cheng et al., 2006; Weng et al., 2010, 2012), were also implemented to extract details of the conformational transition in one functional rotating step (Schulz et al., 2010, 2011; Vargiu et al., 2011). In this work, we performed targeted MD simulations to investigate the conformational transition with different number of binding substrates. Binding of one or two molecules of doxorubicin show similar concerted conformational changes in the translocation pathway and the dissociation of substrate from the DBP. A close correlation was observed between side chain reorientations of Phe628 and Tyr327 and substrate dissociation in both systems. Binding of a second substrate evidently facilitates the conformational changes in the translocation pathway as well as the dissociation of the DBP-bound substrate, explaining the positive cooperativity in substrate transportation.

## Materials and methods

### System setup

The crystal structure of AcrB (PDBID: 2GIF) was used as the starting structure of all simulations. In the crystal structure, residues 1034–1049 are missing in chain A and C, and residues 1046–1049 are missing in chain B. To keep the 3-fold symmetry of the protein, we truncated the residues 1034–1045 in chain B. Since no substrate was cocrystallized in this structure, one doxorubicin was docked into the DBP of B monomer according to the AcrB-doxorubicin complex structure (PDBID: 2DR6). In the simulations with two substrates, the second doxorubicin was docked into the PBP of A monomer by using Autodock (Morris et al., 2009). Default parameters were used for the docking procedure and the center structure of the largest cluster was selected for the simulations.

The AcrB-doxorubicin complex was then buried into a pre-equilibrated palmitoyloleoylphosphatidylethanolamine (POPE) bilayer consisting of 512 lipid molecules following the “shrinking” method (Kandt et al., 2007). Additional 10 lipid molecules (5 lipids in the outer leaflet and 5 in the inner leaflet) were manually placed in the central cavity enclosed by the three TM domains. The number of lipids was estimated by the area of the cavity calculated by Hole (Smart et al., 1996) divided by the area per lipid for POPE (59 Å^2^) (Rappolt et al., 2003). The protein-lipids complex were solvated with a rectangular box of water and neutralized by 42 Na^+^ ions. The box size is 140 × 140 × 160 Å^3^ to keep any atom of protein at least 10 Å away from the edge of the box. The simulation system contains 320,557 atoms in total (Figure 1A).

### MD simulations

All simulations were carried out with the parallel MD package NAMD 2.7 (Phillips et al., 2005) with CHARMM27 force field (Feller et al., 1997; MacKerell et al., 1998, 2004). TIP3P model (Jorgensen et al., 1983) was used for water molecules. The CGenFF force field parameters of doxorubicin were derived by using the ParamChem tool (Vanommeslaeghe et al., 2010, 2012; Vanommeslaeghe and MacKerell, 2012). All titratable residues were kept in their default protonation states except Asp407 and Asp408. For these two residues, two different protonation schemes were used. In the first scheme, the protonation states were set according to the asymmetric structure of AcrB (Murakami et al., 2006), i.e., Asp407 and Asp408 were deprotonated in the A and B monomers, and protonated in the E monomer. In the second scheme, Asp407 and Asp408 were protonated in the B monomer and deprotonated in the A and E monomers. The system was maintained at 1.01325 bar by using the Nosè-Hoover Langevin piston method (Martyna et al., 1994; Feller et al., 1995) and at 300 K by Langevin dynamics with a damping coefficient of 1.0 ps^−1^. Periodic boundary conditions were employed, and the electrostatic interactions were evaluated using the particle-mesh Ewald (PME) method (Darden et al., 1993) with a cutoff of 12 Å and a grid spacing of 1 Å. The van der Waals interactions were switched at 10 Å and truncated at 12 Å. All bonds involving hydrogen atoms were constrained by the SETTLE algorithm (Miyamoto and Kollman, 1992). A time step of 2 fs was used for the conventional MD simulations, and 1 fs was used for the targeted MD simulations.

The whole system was equilibrated for 2 ns with the protein restrained by harmonic forces (the force constant was set to 1000 kcal/mol/Å^2^), followed by a 4 ns run without any restraint. AcrB was stable in the 4 ns NPT run with its C_α_ root-mean-square deviation (RMSD) relative to the initial crystal structure fluctuating around 2.2 Å in the last 2 ns. The final structure of the unbiased MD run was used as the initial structure of the targeted MD simulations. The target structure was obtained by setting each monomer to the state of the next functional rotating step, i.e., ABE→BEA.

Targeted MD method propels a known initial structure to a known target structure by using an external potential (Schlitter et al., 1993). The potential decreases the RMSD of the system relative to the target structure toward a preset value at each time step. The potential can be described as:

$$U_{TMD}=\frac{1}{2}\frac{k}{N}{\left[RMSD(t)-RMSD*(t)\right]}^{2}$$

where *RMSD(t)* is the instantaneous best-fit RMSD of the current coordinates to the target coordinates, RMSD^*^(t) is the preset RMSD value for the current time step, *k* is the force constant and *N* is the number of targeted atoms. The external forces were casted on all heavy atoms of AcrB. A tclForce script was used to perform mass-weighted targeted MD simulations. To assess the influence of parameters on the results, we performed a series of simulations using different force constants (*k/N* = 1, 2, and 3 kcal/mol/Å^2^) and different simulation times (500 ps and 1 ns) (Table S1, Supplemental Data).

### Data analyses

The radius of gyration (*R_g_*) of the DBP is defined by all the atoms in the residues 628, 610, 136, 178, 615, 617, 277, 626, 620, 176, and 612 which lies in the vicinity of DBP:

$$R_{g}\;=\;\left(\sum_{i\;=\;1}^{n}\omega \left(i\right){\left(r\left(i\right)-r_{mc}\right)}^{2}\right)/\left(\sum_{i\;=\;1}^{n}\omega \left(i\right)\right)$$

where *r(i)* is the position of the *i*th atom, *ω (i)* is the mass weight and *r_mc_* is the weighted center of the selected atoms. The distance between Gln124 & Tyr758 is defined as the distance between the C_α_ atoms of the two residues. All data analysis and molecular structure visualization were conducted by VMD (Humphrey et al., 1996).

## Results

We employed targeted MD simulations to acquire an atomistic view of the conformation changes of AcrB in one functional rotating step. The conformational changes were studied with one doxorubicin bound in the DBP of B monomer (denoted as **dox** hereafter), or with two doxorubicins bound in the DBP of B monomer and in the PBP of A monomer, respectively (denoted as **2dox** hereafter). Three parallel trajectories started with different initial velocities were produced for each system. The results were found to be insensitive to the selection of force constants or time lengths (Table S1, Supplemental Data, see below for details). In the following, we will first introduce the collective and local conformational changes in the translocation pathway and the association of these changes with doxorubicin movement. Then we focus on the effect of the presence of the second doxorubicin by comparing the trajectories of the **dox** and **2dox** systems. For simplicity, we use the targeted MD simulation time to describe the conformational transition events in the text, but it is worth noting that the simulation time only denotes the relative progress of the targeted MD trajectories and is not necessarily proportional to actual time.

### Concerted conformational changes in the translocation pathway of B monomer

At the beginning of the simulation, large-scale conformational changes were observed in the porter domain of the B monomer (Figure 1B). In all three trajectories of the **dox** system, the C_α_ RMSDs of the porter domain decreased linearly relative to the targeted structure in the first 330 ps, after which the RMSD profiles fluctuated around 1.7 Å till the end of the simulations (Figure 1C). The variations of the porter domain are largely resulted from the relative motions between the subdomains. The motions reduced the distance between the mass centers of PC1 and PC2 subdomains from 26.6 to 22.8 Å in 330 ps (Figure 1D), closing the PC1–PC2 cleft and occluding the translocation pathway toward the entrance (Figure 1B). On the other hand, the DBP shrunk from ~100 ps, with the *R_g_* decreasing till the end of the simulations (Figure 1E). The decline of *R_g_* indicates contraction of the middle part of the translocation pathway which would contribute to the dissociation of substrate (see below). During this period, the distance between residues Gln124 and Tyr758, which constrict the exit of drug extrusion (Yao et al., 2013) (Figure 1B) increased from 8.7 to 13.1 Å in about 180 ps (Figure 1F), resulting in the opening of the exit. It is worth noting that the timing of these conformational changes obviously overlap with each other, with the exit gate opening at 155–340 ps (Figure 1F), the PC1/PC2 cleft closing at 50–330 ps (Figure 1D) and the DBP shrinking at 75–500 ps (Figure 1E), indicating concerted conformational motions of the translocation pathway in the first 330 ps of the simulations.

### Dissociation of doxorubicin from the DBP

As the concerted transmutation of the translocation pathway started in the first 155 ps, the substrate kept stably bound inside the DBP through hydrophobic and π −π interactions with the protein (Figure S1, Supplemental Data). The binding mode in the simulations is slightly different from the two known binding modes presented in crystal structures (Murakami et al., 2006; Eicher et al., 2012). The position of the amino-sugar moiety was almost invariant, whereas the aglycone moiety showed a new posture by extending directly toward Phe628 rather than pointing toward Phe610 (Eicher et al., 2012) or Phe617 (Murakami et al., 2006) in the crystal structures (Figure S1, Supplemental Data). The diversity of binding mode could be attributed to the differences in the local configuration of the DBP in different crystal structures (see Methods). Similar structural dependence of binding mode was also reported for chloramphenicol in the DBP of AcrB (Vargiu and Nikaido, 2012).

**Figure 2:**
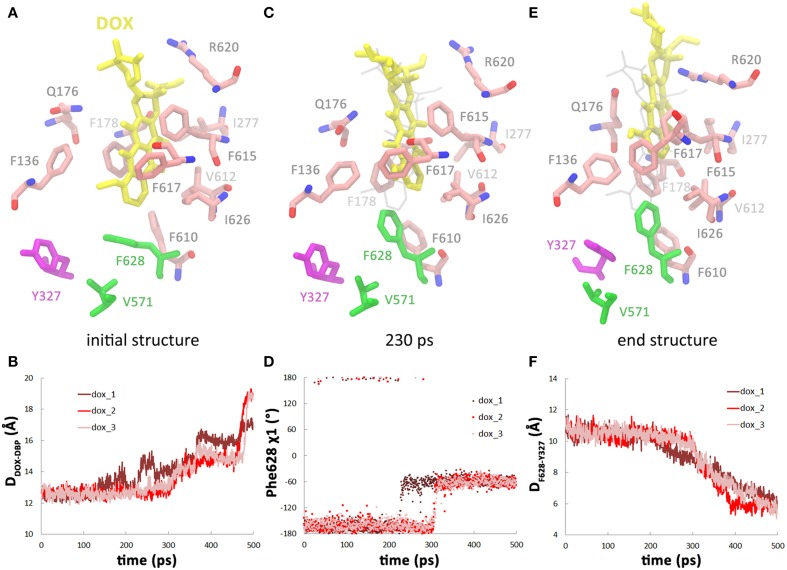
**Dissociation of doxorubicin from the DBP in the dox system. (A)** Close view of doxorubicin (yellow) bound in DBP in the initial structure. Residues lining the DBP are colored by atom type (C: salmon, O: red, N: blue), except Phe628, Val571, and Tyr327, which are highlighted by green and purple. **(B)** Variations of the distance between doxorubicin and the DBP with simulation time. **(C)** Snapshot of doxorubicin in DBP at 230 ps in one of the trajectories when Phe628 reoriented and pointed toward the substrate. **(D)** Variations of χ 1 angle of Phe628 along the simulation trajectories. **(E)** Close view of doxorubicin and the residues lining DBP at the end of the simulation when Tyr327 inserted in between Phe628 and Val571. **(F)** Variations of the distance between the mass center of Tyr327 side chain and the C_α_ atom of Phe628.

The stable interactions between doxorubicin and DBP were disrupted at 225–300 ps as doxorubicin detached from the binding site. The dissociation of doxorubicin was monitored by the distance between the mass center of doxorubicin and the C_α_ atom of Phe628 which lies at the bottom of the DBP (Figure 2A). Two of the trajectories exhibit very similar profiles of substrate motions. The doxorubicin initially stayed in the DBP with the distance fluctuating around 12.5 Å, and detached from the binding site after 300 ps as the distance increased (Table 1, Figure 2B, pink and red lines). In another trajectory, more evident fluctuations were observed before dissociation, such as the peak at 225 ps on the profile (Figure 2B, brown line). After 275 ps, doxorubicin became fully dissociated from the DBP. At the end of the simulations, a displacement of 5–7 Å toward the exit could be observed and the dissociation is irreversible, as verified by the extended 2-ns simulations with all restraining forces removed (Figure S2, Supplemental Data). Similar extrusion process was also observed by Vargiu et al. using targeted MD simulations (Vargiu et al., 2011).

**Table 1:** **Conformational changes associated with the dissociation of doxorubicin**.

**System^a^**	**Trajectory**	**Time of doxorubicin dissociation (ps)^b^**	**Time of Phe628 side chain reorientation (ps)^c^**	**Time of Tyr327 side chain insertion (ps)^d^**
**dox**	1	225, 275	225	235–500
	2	300	300	300–395
	3	300	300	300–470
**2dox**	1	200	200	200–400
	2	225	225	225–370
	3	190	190	190–420

a **dox** denotes the simulation system with only one doxorubicin bound in DBP of B monomer, **2dox** denotes the simulation system with a second doxorubicin bound in PBP of A monomer.

b Time when doxorubicin detached from the binding pocket. Temporary dissociation leads to more than one dissociation time.

c Time when Phe628 reoriented its side chain and pointed toward the substrate.

d Time period of the decreasing of the distance between Tyr327 side chain and Phe628 C_α_.

### Correlation between the conformational changes in the DBP and doxorubicin dissociation

Further inspection of the trajectories show that the dissociation process of doxorubicin is closely related to the conformational changes of the DBP, especially the side chain orientation of Phe628. The benzyl group of Phe628 originally pointed toward the TM domain at the beginning of the simulations, characterized by the χ 1 angle of about −160° (Figure 2A), and then it switched to a new orientation with the χ 1 angle at about −60° (Figures 2A,C,D). The reorientation of Phe628 side chain occurred at 300 ps in two of the trajectories (Figure 2D, pink and red lines) and at 225 ps in the other (Figure 2D, brown line), well consistent with the departure time of doxorubicin (Table 1). The close correlation between the two events can be rationalized by the steric hindrance between the bulky side chain of Phe628 and the doxorubicin at binding site (Figure 2C), which pushes the substrate from the binding pocket. The importance of Phe628 for substrate dissociation was previously reported by biochemical studies, in which substitution of the bulky side chain reduced the resistance to doxorubicin and other substrates (Husain and Nikaido, 2010; Nakashima et al., 2011).

After the reorientation of Phe628, local conformational rearrangement was observed. The space originally occupied by the benzyl group of Phe628 and the isopropyl group of Val571 (Figures 2A,C) was subsequently filled by the phenol group of Tyr327, which is several residues preceding TM2 helix of the TM domain (Figure 2E). Interestingly, the rearrangement started almost simultaneously with the reorientation of the Phe628 side chain (Figure 2F, Table 1). This implies a functional role of Tyr327 in stabilizing the reorientated conformation of Phe628.

To examine possible influence of TM domain protonation state on substrate dissociation, we produced targeted MD trajectories for AcrB with different protonation state (Table S1, Supplemental Data). It turns out that these trajectories demonstrated very similar features such as substrate dissociation, side chain reorientation of Phe628 and Tyr327 (Figure S3, Table S1, Supplemental Data), indicating that the current simulation protocol is insensitive to the changes in protonation state. Different simulation time range (500 ps or 1 ns) and different force constant (*k/N* = 1, 2, or, 3 kcal/mol/Å^2^) were also tested. These factors do not disturb the correlation between conformational changes in the DBP and substrate dissociation, either (Table S1, Supplemental Data).

### Binding of the second doxorubicin accelerates the conformational changes in the translocation pathway

**Figure 3:**
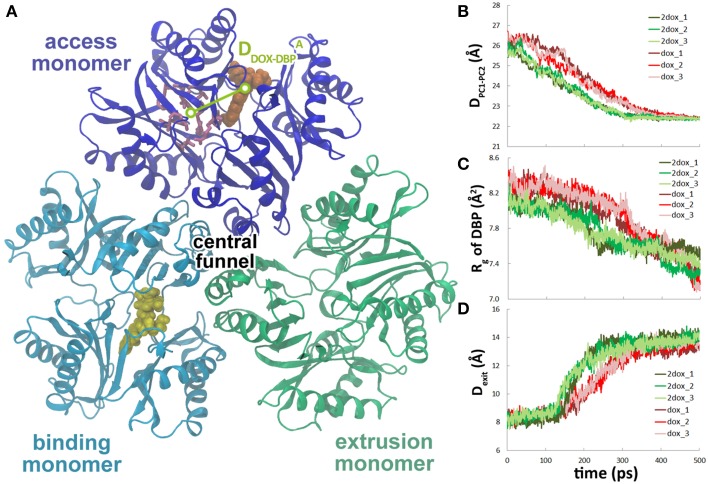
**The second substrate binding at A monomer accelerates the conformational changes of B monomer. (A)** Top view of the porter domains with two doxorubicins (yellow and orange) bound in the initial structure of the **2dox** system. The distance between doxorubicin and DBP in A monomer is indicated as D^A^_DOX−DBP_. **(B)** Comparison of the variations of the distance between the mass centers of PC1 and PC2 subdomains of B monomer in **dox** and **2dox** systems. **(C)** Comparison of the variations of R_*g*_ of the DBP of B monomer in **dox** and **2dox** systems. **(D)** Comparison of the variations of the Gln124-Tyr758 inter-residue distance at the exit region of B monomer in **dox** and **2dox** systems.

To study the effect of multi-substrate binding on the conformational transition, a second doxorubicin was docked into the PBP of A monomer (see Methods) to build the **2dox** system (Figure 3A). The position of the docked substrate closely resembles the binding mode of PC1-proximal doxorubicin (Figure S4, Supplemental Data) observed in the AcrB-doxorubicin complex structure (Eicher et al., 2012). The presence of the second doxorubicin affects the conformational changes in the translocation pathway in several aspects. First, the closing motion of the PC1/PC2 cleft was antedated, illustrated by the ~70 ps earlier decreasing period of the inter-domain distance in the **2dox** system than that in the **dox** system (Figure 3B). Secondly, the conformational changes in the DBP were moved ahead as the *R_g_* decreased to 7.8 Å at about 200 ps in the **2dox** system, instead of at 300 ps in the **dox** system (Figure 3C). Finally, the opening motion of the exit was accelerated as the distance between Gln124 and Tyr758 exceeded 13 Å at 220 ps, rather than at 300 ps in the **dox** system (Figure 3D). It is worth noting that the **dox** and **2dox** systems share very similar initial and final structures at the PC1/PC2 cleft, the DBP and the exit gate (Figures 3B–D), so that the external forces casted on these regions are generally equivalent in both systems (see Methods). The systematic antedate of these events indicates that the conformational changes in the translocation pathway are evidently accelerated by the binding of the second doxorubicin at A monomer.

### Binding of the second doxorubicin facilitates the dissociation of doxorubicin

**Figure 4:**
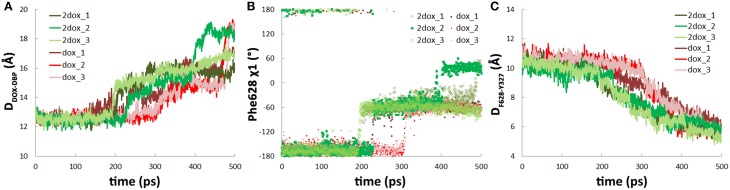
**The second substrate binding at monomer A accelerates the dissociation of doxorubicin from the DBP in monomer B. (A)** Comparison of the variations of the distance between doxorubicin and the DBP of monomer B in **dox** and **2dox** systems. **(B)** Comparison of the variations of χ 1 angle of Phe628 in **dox** and **2dox** systems. **(C)** Comparison of the variations of the distance between the mass center of Tyr327 side chain and the C_α_ atom of Phe628 in **dox** and **2dox** systems.

Along with the antedated conformational changes in the translocation pathway, substrate dissociation in the **2dox** system was also earlier than that in the **dox** system. In the three trajectories, irreversible dissociation occurred at 200, 230, and 190 ps, respectively (Figure 4A, Figure S5, Supplemental Data), about 70 ps earlier than that of the **dox** system on average. Along with substrate dissociation, the change of Phe628 χ 1 angle from −160 to −60° and the insertion of Tyr327 in between Phe628 and Val571 were also moved ahead to about 200 ps (Figures 4B,C). Similar with the **dox** system, there is also a close correlation between substrate dissociation and side chain reorientation of Phe628 and Tyr327 (Table 1) and the trend is kept with varied simulation time range or force constant (Table S1, Supplemental Data), indicating that the **2dox** system shares the same mechanism of doxorubicin dissociation in B monomer with the **dox** system.

### Conformational changes in AcrB does not promote the transportation of the second doxorubicin

**Figure 5:**
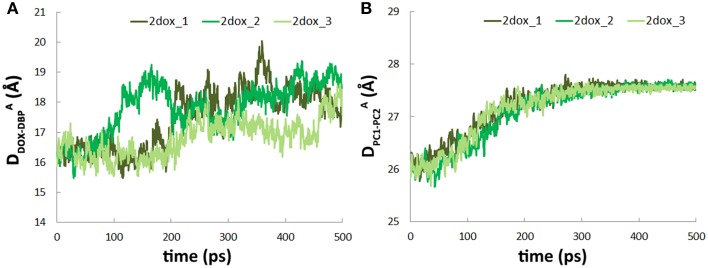
**Conformational transition of A→B does not facilitate substrate translocation from PBP to DBP. (A)** Variations of D^A^_DOX−DBP_ along the simulation trajectories. **(B)** Variations of the distance between the mass centers of PC1 and PC2 subdomains in A monomer.

The movement of doxorubicin in the PBP of A monomer during the functional rotation was also examined (Figure 3A). In contrast to the displacement toward the exit observed for the DBP-bound doxorubicin in B monomer, the PBP-bound doxorubicin in A monomer moved in an opposite direction, further away from the DBP by about 2 Å at the end of the simulations (Figure 5A). The slight retrogression of the substrate could be attributed to the inter-domain motion between PC1 and PC2 subdomains. The PC1/PC2 cleft opened by about 1.5 Å when A monomer evolved toward the B state following the functional rotating mechanism (Figure 5B). The opening motion may weaken the interactions between AcrB and doxorubicin, and leads to the movement of substrate toward the entrance.

## Discussion

The functional rotating mechanism based on the asymmetric structure of AcrB assumes that each monomer cycles among the three conformational states (A, B, and E) concertedly, facilitating the unidirectional translocation of the substrate toward the outer membrane protein TolC. In this work, we simulated one functional rotating step of AcrB (ABE→BEA) in the context of one or two bound substrates using targeted MD simulations, and provided an atomic spatiotemporal view of the conformational changes and substrate movement.

The targeted MD trajectories demonstrated that the simulation started with conformational changes in the translocation pathway, involving a set of concerted motions such as the closing of the PC1/PC2 cleft (Figure 1D), the shrinking of the DBP (Figure 1E), and the opening of the exit gate (Figure 1F). In the following 200 ps, however, doxorubicin in DBP did not show evident motions until the dissociation from DBP occurred at 225–300 ps. Therefore, the conformational changes in the translocation pathway are the prerequisite and the driving force for substrate dissociation. Specifically, it has been identified that the side chain reorientation of Phe628 is mainly responsible for substrate dissociation (Figure 2). The importance of Phe628 for substrate transportation is supported by previous biochemical experiments, in which removal of the bulky side chain of Phe628 evidently reduced the resistance to doxorubicin and other substrates of AcrB (Husain and Nikaido, 2010; Nakashima et al., 2011). Similar protein-driven substrate movement was also observed by A. Vargiu et al. using the same method (Schulz et al., 2010; Vargiu et al., 2011) though the atomistic details of protein-substrate interaction are slightly different. Due to the limited simulation time in this work, we did not observe the entire extrusion process of the substrate. Hundreds of nanoseconds or more may be needed for the substrate to overcome the barriers on its way to the central funnel (Ruggerone et al., 2013; Yao et al., 2013).

The functional rotating mechanism of AcrB assumes that the conformational transitions of the three monomers occur in a concerted way, implicating cooperativity in substrate translocation. Kinetic studies of substrate transport of AcrB in intact cells identified positive cooperativities in the efflux of various cephalosporins (Nagano and Nikaido, 2009) and penicillins (Lim and Nikaido, 2010). Efflux could also be stimulated by different kinds of substrates, including solvents (Kinana et al., 2013). The positive cooperativity in substrate efflux could be explained by the simultaneous binding of substrates to PBPs in the B or A monomers. Crystal structures of the AcrB-substrate complex revealed simultaneous substrate binding at DBP of B monomer and at PBP of A monomer (Nakashima et al., 2011; Eicher et al., 2012), implying that the substrate in PBP of A monomer more likely plays a role in stimulating the efflux. Simulations in this study provide the direct evidences that substrate binding at PBP of A monomer indeed stimulates substrate dissociation in B monomer through accelerating the conformational transitions according to the functional rotating mechanism. We noticed that the conformational changes were overall accelerated by the substrate in A monomer, not just the local changes in DBP (Figure 3). This is in line with the observation that the substrate would not dissociate from the DBP until most of the conformational changes completed during the functional rotation. Therefore, the substrate dissociation is allosterically regulated by the substrate binding in the neighboring monomer. Another possibility we could not rule out, however, is that ligand binding at PBP of the same monomer, i.e., in the B monomer, may also stimulate the substrate extrusion, although the corresponding intermediate state has not been detected in crystallography studies. Yet another mechanism of the positive cooperativity in substrate extrusion proposed by A. Kinana et al. is the direct interference of the stimulator with the substrate through competitive binding at the same DBP of B monomer (Kinana et al., 2013). It is worth noting that in the same study, decreases of Hill coefficients were observed in the presence of the stimulators (Kinana et al., 2013), which supports the allosteric mechanism with stimulators bound at different sites.

Another implication for the observation that substrate binding at A monomer promotes the protein conformational changes is that the functional rotation of AcrB trimer is partly energized by ligand binding in addition to the proton motive force. The accelerated conformational transition indicates that the free energy barrier of this process is lowered upon ligand binding. In other words, substrates are not only passively pumped by the molecular machine of AcrB, but also actively regulate the functional rotation of AcrB trimer. Recent combined crystallography and MD simulation study also suggested that drug binding is the driving force in the A to B (or L to T) transition (Mishra et al., 2014).

An unexpected observation in this work is that the functional rotation did not squeeze the PBP-bound substrate toward DBP during the A–B transition, rather it moved away toward the entrance. This might be attributed to the slight opening of the PC1–PC2 cleft upon A–B transition (Figure 5), suggesting that substrate translocation from PBP to DBP may require temporary closure of the cleft. In line with this, partially closed conformations of the cleft have been observed in monomer A by unbiased substrate-free MD simulations (Fischer and Kandt, 2013). An intermediate state with PC1–PC2 cleft closed was also identified in another RND transporter ZneA (Pak et al., 2013). It is most likely that during the functional rotation, a similar intermediate state between A and B yet identified also exists in AcrB to prevent the back diffusion of the substrate.

Although our simulation was conducted on isolated AcrB, the RND transporter functions together with the periplasmic adaptor protein AcrA and outer membrane protein TolC as a tripartite assembly, and AcrA-Mg^2+^ has been reported to strongly accelerate the extrusion of substrate (Zgurskaya and Nikaido, 1999b). These findings raise an issue of whether the acceleration effect of multi-substrate binding observed in our simulation would still work after the association of AcrA-Mg^2+^. The crystal structure of heavy-metal efflux complex CusBA (Su et al., 2011), a homology of AcrAB, and the recently reported pseudo-atomic structure of AcrAB-TolC complex (Du et al., 2014) provide a valuable insight into the acceleration effect of AcrA-Mg^2+^. The structures show that the adaptor protein (CusB or AcrA) forms interactions with the TolC-docking domain and the subdomains PN2, PC1, and PC2 of the RND transporter. A noteworthy feature of the interactions between adaptor protein and RND transporter is that the subdomain PC2 forms much weaker contacts with the adaptor protein than PN2 and PC1 do. Only two residues on PC2 (Arg705 and Leu714) are located within 4 Å of the adaptor protein and they form weak van der Waals interactions. The binding of adaptor protein may restrain the motions in PN2 and PC1, but is less likely to disturb the motions of PC2 and the relative motions between PC1 and PC2. Since the second substrate in monomer A binds between the PC1 and PC2 subdomains, the adaptor protein may have little influence on the behavior of the second substrate and the accelerated conformational changes brought by its binding. The acceleration effects of AcrA-Mg^2+^ and multi-substrate binding may operate simultaneously when AcrB functions. It is worth noting, however, that the above notions are largely derived based on the static structures of CusBA and AcrAB-TolC complexes. Further studies, especially those concerning dynamic properties, are still required to provide a fuller understanding on the molecular mechanism of the acceleration effects.

### Conflict of interest statement

The authors declare that the research was conducted in the absence of any commercial or financial relationships that could be construed as a potential conflict of interest.
