# Supplementary material for: Substrate binding accelerates the conformational transitions and substrate dissociation in multidrug efflux transporter AcrB
Source: Front Microbiol. 2015 Apr 13;6:302. doi: 10.3389/fmicb.2015.00302 (PMC4394701; doi:10.3389/fmicb.2015.00302)
Supplement: Supplementary file 1 [file Table1.DOCX]

**Table S1.** Conformational changes associated with the dissociation of doxorubicin in trajectories with different simulation times and force constants. Definitions of the events are the same as Table 1.

| System^a^ | Simulation time | Force constant k_atom_ | Number of trajectories | Time of doxorubicin dissociation | Time of Phe628 side chain reorientation | Time of Tyr327 side chain reorientation |
| --- | --- | --- | --- | --- | --- | --- |
|  | (ps) | (kcal·mol^-1^Å^-2^) |  | (ps) | (ps) | (ps) |
| **dox** | 500 | 1.0 | 3 | 225, 275 | 225 | 235-500 |
|  |  |  |  | 300 | 300 | 300-395 |
|  |  |  |  | 300 | 300 | 300-470 |
| **2dox** | 500 | 1.0 | 3 | 200 | 200 | 200-400 |
|  |  |  |  | 225 | 225 | 225-370 |
|  |  |  |  | 190 | 190 | 190-420 |
| **dox**^ori^ | 500 | 1.0 | 3 | 310 | 300 | 295-385 |
|  |  |  |  | 245 | 240, 260 | 250-370 |
|  |  |  |  | 225 | 235, 260 | 210-385 |
| **dox** | 500 | 2.0 | 3 | 280 | 290 | 280-385 |
|  |  |  |  | 335 | 330 | 260-390 |
|  |  |  |  | 300 | 290 | 275-370 |
| **2dox** | 500 | 2.0 | 3 | 315 | 310 | 295-430 |
|  |  |  |  | 155 | 60, 110, 160 | 245-365 |
|  |  |  |  | 165 | 165 | 135-390 |
| **dox** | 500 | 3.0 | 1 | 210, 250 | 215, 260 | 265-375 |
| **dox** | 1000 | 1.0 | 1 | 450, 540 | 415 | 420-800 |
| **2dox** | 1000 | 1.0 | 1 | 400 | 350 | 380-740 |
| **dox** | 1000 | 2.0 | 1 | 580 | 575 | 540-785 |
| **dox** | 1000 | 3.0 | 1 | 480 | 485 | 535-755 |

^a^ Residues Asp407 and Asp408 were protonated in the B monomer and deprotonated in the A and E monomers. ^ori^ Residues Asp407 and Asp408 were deprotonated in A and B monomers, and protonated in the E monomer.
