# Supplementary material for: Substrate binding accelerates the conformational transitions and substrate dissociation in multidrug efflux transporter AcrB
Source: Front Microbiol. 2015 Apr 13;6:302. doi: 10.3389/fmicb.2015.00302 (PMC4394701; doi:10.3389/fmicb.2015.00302)
Supplement: Supplementary file 2 [file Image1.PDF]

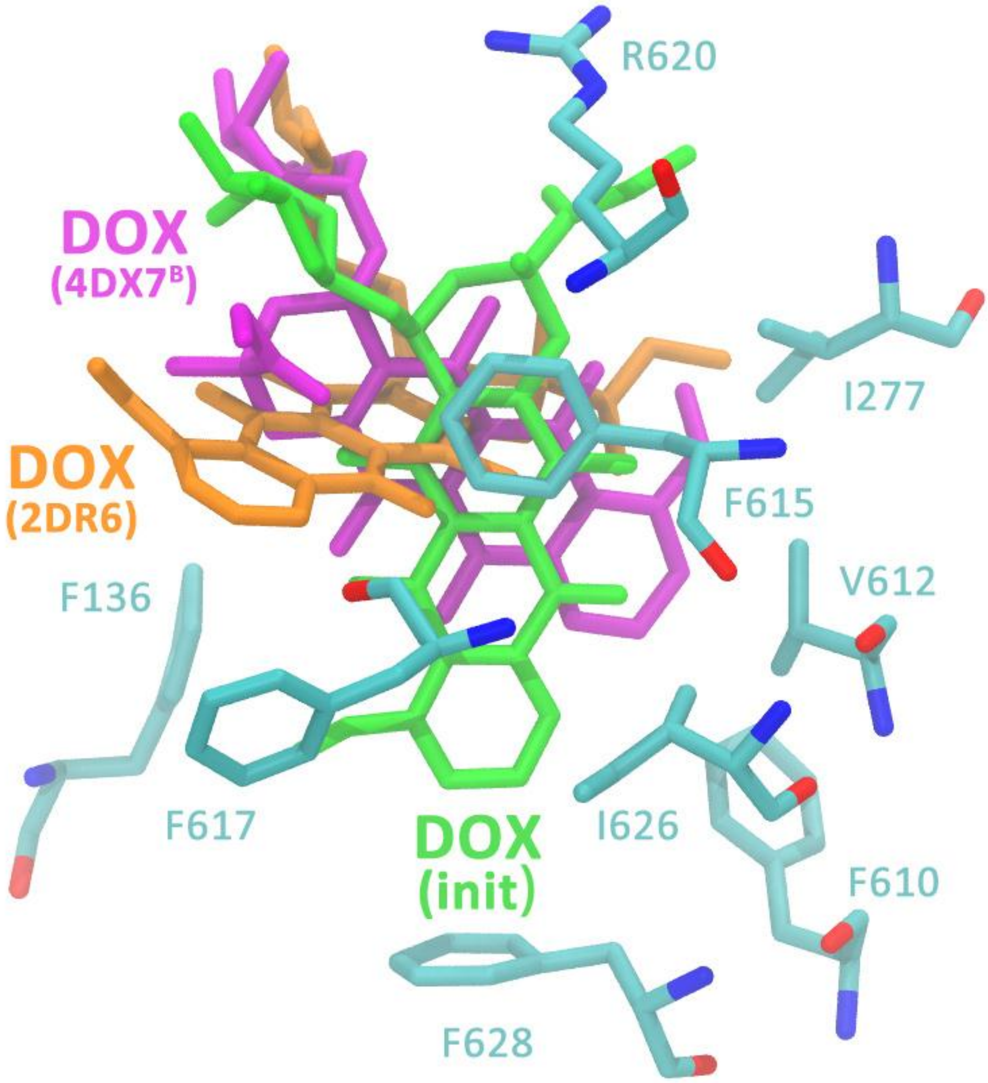

Figure S1. Comparison of the binding mode in the DBP of the initial structure (green) and those in the crystal structures (magenta and golden). Residues lining the DBP are colored in cyan.
