# Supplementary material for: Substrate binding accelerates the conformational transitions and substrate dissociation in multidrug efflux transporter AcrB
Source: Front Microbiol. 2015 Apr 13;6:302. doi: 10.3389/fmicb.2015.00302 (PMC4394701; doi:10.3389/fmicb.2015.00302)
Supplement: Supplementary file 3 [file Image2.PDF]

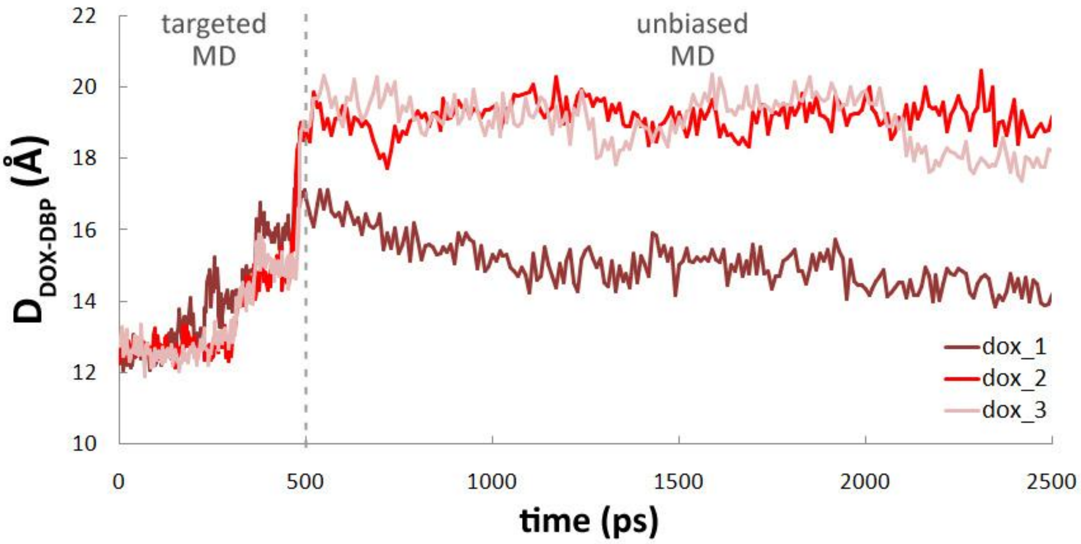

Figure S2. Variations of the distance between doxorubicin and the DBP during the 500-ps targeted MD simulation (k/N = 1 kcal/mol/$Å^2$) and the following 2-ns unbiased MD simulation of the **dox** system. The DBP is represented by the C_α atom of Phe628.
