# Supplementary material for: Substrate binding accelerates the conformational transitions and substrate dissociation in multidrug efflux transporter AcrB
Source: Front Microbiol. 2015 Apr 13;6:302. doi: 10.3389/fmicb.2015.00302 (PMC4394701; doi:10.3389/fmicb.2015.00302)
Supplement: Supplementary file 4 [file Image3.PDF]

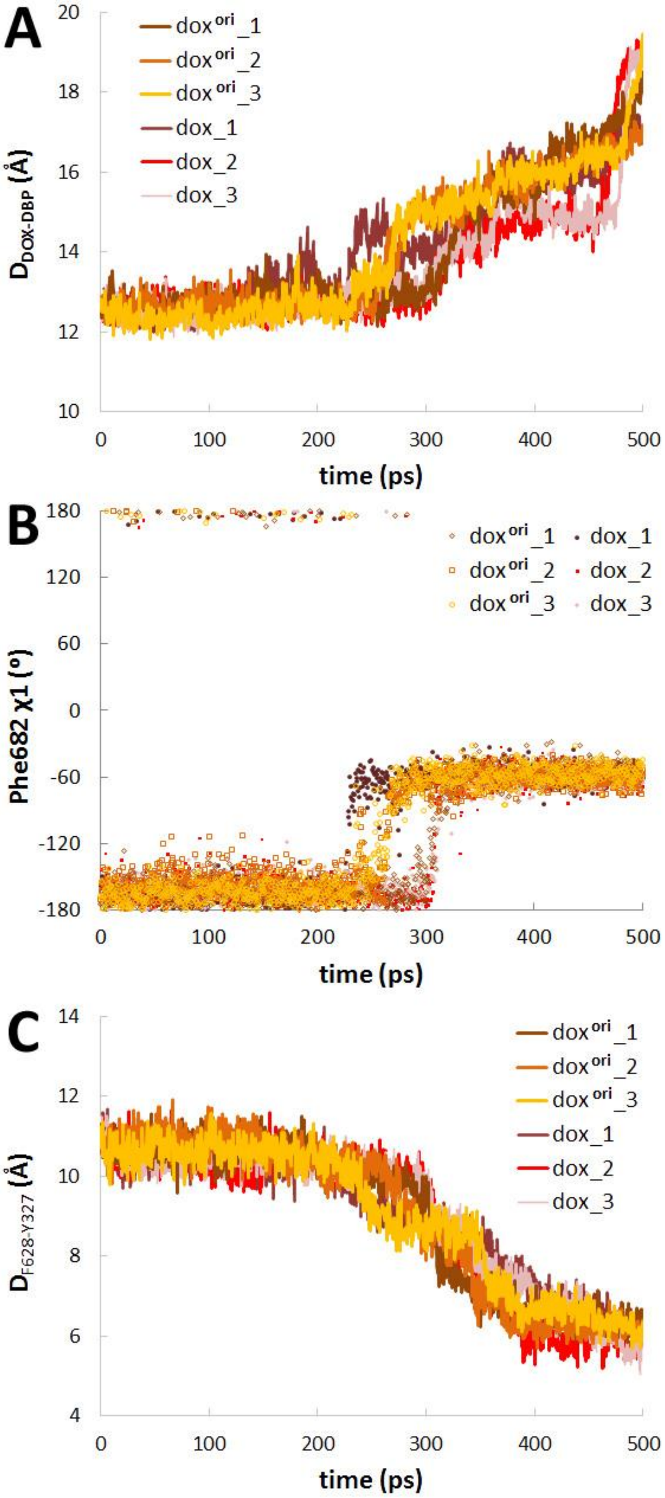

Figure S3. Comparison of the dissociation of doxorubicin from the DBP in different protonation states of Asp407 and Asp408 (See the footnotes of Table S1 for details). (A) Variations of the distance between doxorubicin and the DBP with simulation time. (B) Variations of $\chi 1$ angle of Phe628. (C) Variations of the distance between the mass center of Tyr327 side chain and Phe628 C_α atom.
