# Supplementary material for: Substrate binding accelerates the conformational transitions and substrate dissociation in multidrug efflux transporter AcrB
Source: Front Microbiol. 2015 Apr 13;6:302. doi: 10.3389/fmicb.2015.00302 (PMC4394701; doi:10.3389/fmicb.2015.00302)
Supplement: Supplementary file 5 [file Image4.PDF]

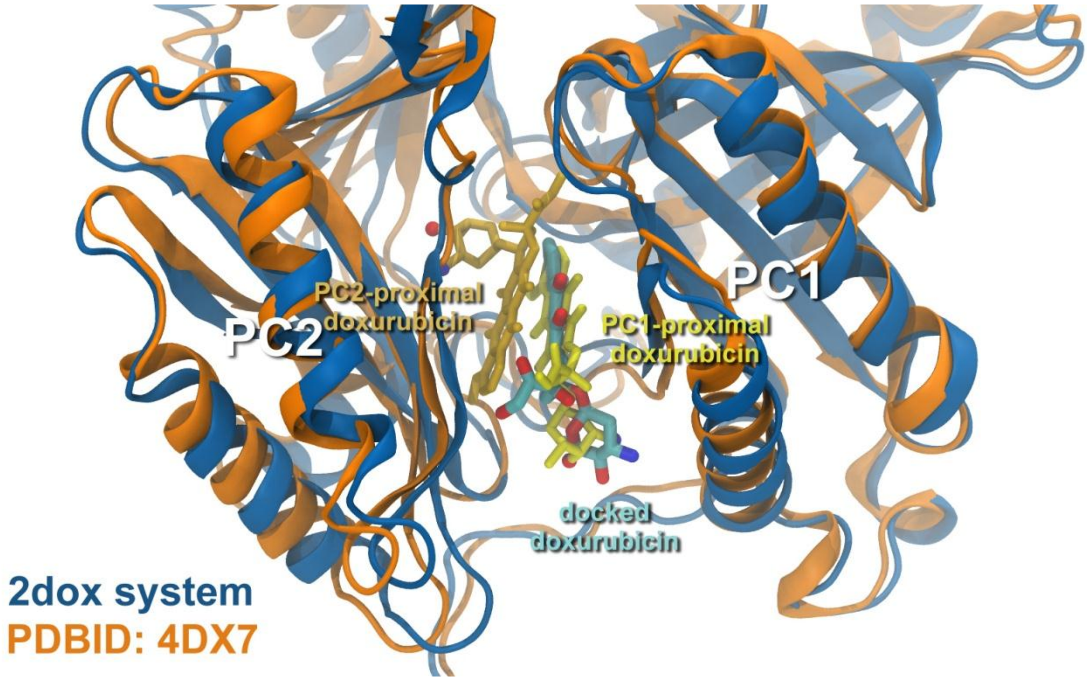

Figure S4. Superimposition of the docked doxorubicin (cyan) in the PBP of **2dox** simulation system and the two doxorubicin molecules (yellow and dark yellow) sandwich in the crystal structure (PDBID: 4DX7). The doxorubicin near PC1 subdomain in crystal structure is denoted as PC1-proximal and that near PC2 as PC2-proximal.
